# Racial disparity in pathophysiologic pathways of preterm birth based on genetic variants

**DOI:** 10.1186/1477-7827-7-62

**Published:** 2009-06-15

**Authors:** Ramkumar Menon, Brad Pearce, Digna R Velez, Mario Merialdi, Scott M Williams, Stephen J Fortunato, Poul Thorsen

**Affiliations:** 1 The Perinatal Research Center, Nashville, Tennessee, USA; 2 Department of Epidemiology, Rollins School of Public Health, Emory University, Atlanta, Georgia, USA; 3 Department of Reproductive Health and Research, World Health Organization, Geneva, Switzerland; 4 Graduate Division of Biological and Biomedical Sciences, Dept. of Psychology, Emory University, Atlanta, Georgia, USA; 5 Miami Institute of Human Genomics and Dr. John T. Macdonald Foundation Department of Human Genetics, University of Miami, Miami, Florida, USA; 6 Center for Human Genetics Research, Vanderbilt University, Nashville, Tennessee, USA; 7 Members of Preterm birth Research International Center for Excellence (PRiCE) at Emory University, Atlanta, Georgia, USA

## Abstract

**Objective:**

To study pathophysiologic pathways in spontaneous preterm birth and possibly the racial disparity associating with maternal and fetal genetic variations, using bioinformatics tools.

**Methods:**

A large scale candidate gene association study was performed on 1442 SNPs in 130 genes in a case (preterm birth < 36 weeks) control study (term birth > 37 weeks). Both maternal and fetal DNA from Caucasians (172 cases and 198 controls) and 279 African-Americans (82 cases and 197 controls) were used. A single locus association (genotypic) analysis followed by hierarchical clustering was performed, where clustering was based on p values for significant associations within each race. Using Ingenuity Pathway Analysis (IPA) software, known pathophysiologic pathways in both races were determined.

**Results:**

From all SNPs entered into the analysis, the IPA mapped genes to specific disease functions. Gene variants in Caucasians were implicated in disease functions shared with other known disorders; specifically, dermatopathy, inflammation, and hematological disorders. This may reflect abnormal cervical ripening and decidual hemorrhage. In African-Americans inflammatory pathways were the most prevalent. In Caucasians, maternal gene variants showed the most prominent role in disease functions, whereas in African Americans it was fetal variants. The IPA software was used to generate molecular interaction maps that differed between races and also between maternal and fetal genetic variants.

**Conclusion:**

Differences at the genetic level revealed distinct disease functions and operational pathways in African Americans and Caucasians in spontaneous preterm birth. Differences in maternal and fetal contributions in pregnancy outcome are also different between African Americans and Caucasians. These results present a set of explicit testable hypotheses regarding genetic associations with preterm birth in African Americans and Caucasians

## Background

Preterm birth (< 37 weeks of gestation) rates vary considerably between racial groups in the United States. The rate is 18.4% in non-Hispanic African-Americans and 11.7% in non-Hispanic Caucasians in 2005 [1]. The African American population has a disproportionate burden of several adverse socioeconomic and environmental factors that have an indisputable connection with PTB [2]. Nevertheless, racial differences in preterm birth cannot be fully explained based on behavioral, psycho-social, or socioeconomic factors [3-5]. Moreover, there are numerous examples in the scientific literature in which racial disparity in disease prevalence is closely associated with genetic variation [6], and genes that regulate immune and hematological functions are over-represented among these, presumably reflecting adaptation of ancestral populations to geographically-restricted pathogens [7].

Understanding spontaneous preterm birth is difficult due to etiologic and pathophysiologic heterogeneities and racial disparity can further complicates this. Etiologic factors such as infection, stress, placental abruption, uterine distension, and preterm premature rupture of membranes, among others are associated with preterm birth in both races. Redundancy of biochemical pathways and biomarkers of preterm birth regardless of etiology suggests pathophysiologic heterogeneity. One explanation for racial disparity may be genetic variations (polymorphisms) that differ in frequency between races or that provide a different genetic context for particular polymorphisms to function. A review of the literature suggests emerging evidence for gene-gene interactions (epistasis) and gene-environment interactions in preterm birth [8-10], some of which may affect African Americans and Caucasians differently [11]. Candidate gene studies of preterm birth indicate greater than expected (60–80% observed for PTB candidate genes vs. ~42% average across the genome allele and genotype single nucleotide polymorphisms (SNPs) frequencies differences between African-Americans and Caucasians [12,13]. In the context of gene-gene and gene-environment interactions that are becoming increasingly apparent, these differences can affect the ability to detect both single SNP associations and critical pathways between populations, indicating that genetic architectures in the two groups may affect pregnancy outcome [14,15].

In addition to static genetic markers, dynamic biomarkers in amniotic fluid and fetal membranes also reveal significant racial disparity, such as inflammatory marker expression and their concentrations in preterm birth [16,17]. Studies suggest that biomarkers thought to be indicators of preterm birth may not be generalizable. Therefore, a simplified, uniform approach to risk identification and common intervention may not be adequate because of different etiological factors that involve many biomolecular factors. Furthermore, preterm birth can be a function of both maternal and fetal risk factors and their interactions at the genetic and biomarker level making the clinical outcome exceedingly complex.

Based on the evidence generated in our laboratory on genetic (both maternal and fetal) and biomarker data from African-Americans and Caucasians [11-17], we hypothesize that genetic predispositions in preterm birth pathway genes differentially affect biomolecular events in each race and this may explain some of the racial disparity. To assess this, genotyping data generated from a large scale candidate gene study were subjected to pathway analyses, using bioinformatics tools [18,19]. Since genes operate within biological networks, we used the Ingenuity Systems Pathways Analysis Software and Knowledge Base (IPA) to estimate whether the pattern of preterm birth candidate genes points to particular biomolecular networks or disease mechanism(s) in African Americans and Caucasians.

## Methods

This study was approved by the institutional review board at TriStar, the parent company institutional review board of record for Centennial Women's Hospital, Nashville, Tennessee (USA).

Genetic analysis performed on a case control study was used for this report. Mothers between the ages of 18 and 40 were recruited. Gestational age was determined by last menstrual period and corroborated by ultrasound dating. Cases (spontaneous preterm birth) were defined as presence of regular uterine contractions at < 36^0/7 ^weeks gestation (2 contractions/10 minutes with documented cervical changes) followed by delivery. Cases and controls were selected from a Nashville cohort between September 2003 and December 2006. Subjects presented with preterm or term labor were given an opportunity to consent for the study when they matched our inclusion criteria. Subjects with medically indicated preterm births such as multiple gestations, preeclampsia, preterm premature rupture of the membranes, placental previa, infant anomalies, gestational diabetes, poly- and oligohydramnios, and other complications such as surgeries during pregnancies were excluded [11-17]. Controls consisted of women having normal labor and delivery at term (≥ 37^0/7 ^weeks) with no medical or obstetrical complications during pregnancy.

Race was identified by self-report and a questionnaire that traces ethnicity back two generations from the parents [11-17]. Individuals who had more than one racial group in their ancestry were excluded from the study [18-20]. African Americans and Caucasians of non-Hispanic origin was included in this study.

High-throughput candidate gene association studies were performed on 370 maternal (172 cases and 198 controls) and 319 fetal (140 cases and 179 controls) DNA samples from Caucasians and 279 maternal (82 cases and 197 controls) and 243 fetal (65 case and 183 control) samples from African-Americans. The candidate genes are listed in our earlier publications [16,17]. A total of 1536 tag SNPs were screened in 130 preterm birth candidate genes. Genotyping was performed by Illumina's GoldenGate genotyping system [21]. We compared African American cases to Caucasian cases, African-American controls to Caucasian controls for both maternal and fetal DNA.

### Statistical analysis

Statistical tests for differences between case and control and African-American and Caucasian allele and genotype frequencies, measurements of allele frequencies and tests deviations from Hardy Weinberg Equilibrium (HWE) were calculated by the use of Powermarker statistical software [22]. Statistical significance for all single locus tests of association and tests for the deviation from HWE were performed with Fishers Exact tests. Clinical and demographic characteristics between cases and controls were compared using Shapiro-Wilks tests of normality on gravidity, gestational age, gestational weight, APGAR 1, and APGAR 5. All measurements deviated significantly from normality; as a result Mann-Whitney two-sample rank sum tests were used to compare case and control groups. Standard t-tests were used to test whether maternal age differed between cases and controls. χ^2 ^or Fisher's exact tests were used to test for differences in the counts of smokers and non-smokers, income differences, and insurance differences between cases and controls. STATA 9.0 statistical software was used for all analyses of clinical and demographic data and an uncorrected p value ≤ 0.05 was considered significant.

### Ingenuity systems pathway analysis (IPA) and knowledgebase for pathway identification

To examine whether the SNPs found to be putatively-associated with preterm birth in each race mapped to different biological networks and disease functions, we used IPA [23-26]. The gene variants that were statistically significantly-associated with preterm birth (p ≤ 0.05) for each race were entered into the IPA analysis tool. These genes were termed "focus genes." The IPA software was used to measure associations of these molecules with other molecules and disease functions stored in its knowledgebase. The knowledgebase encompasses relationships between proteins, genes, cells, tissues, xenobiotics, and diseases. The information is scientist-curated, updated, and integrated from the published literature and other databases such as OMIM, Gene Ontology, and KEGG.

Disease function categories are among the biological functional ontologies developed by experts across broad domains of biology at IPA. There are 27 higher-order disease and disorder categories in the IPA knowledgebase, and below these higher order categories, there are lower level and specific functions. The IPA functional annotations have integrated GO data [27], but the IPA knowledgebase is more extensive and complete, and therefore uses a different but overlapping terminology. Enrichment of focus genes in higher order disease categories were evaluated by comparing p-values calculated by the IPA software. The p-value for a given function is calculated by considering: 1) the number of identified focus molecules from the user input that participate in that disease and disorder function and, 2) the total number of molecules that are known to be associated with that function in the IPA knowledge base. To derive p-values, the IPA software uses a right-tailed Fisher Exact Tests calculate likelihood that the association between the set of focus genes and a disease function is due to random chance. If a higher-order disease and disorder category contains two or more specific functions reaching statistical significance, the software displays the most statistically significant value on the y axis of the bar graph.

We used the IPA software to identify new molecular network(s) that involve our focus genes. By drawing on its knowledgebase, the IPA software models cellular and molecular networks, including, binding, phosphorylation, proteolysis, nuclear receptor activation, gene transcription, and sub-cellular localization. We mapped focus genes to networks based on their mechanistic associations with each other and partner molecules. The IPA statistical algorithm identifies molecular pathways, involving these focus genes based on their selective interconnectivity with each other and additional molecules stored in the genome-scale knowledgebase. These networks are illustrated and ranked by the software for significance of focus gene enrichment.

## Results

Demographic and clinical data and single locus associations with preterm birth pertaining to this data set has already been published [13,14]. Briefly, as per our definition cases had shorter gestational age. Cases also had lower birth weight and APGAR scores compared to controls, irrespective of race. Frequency of clinical parameters associated with preterm birth such as histologic chorioamnionitis, intraamniotic infection, bacterial vaginosis, and clinical chorioamnionitis (CRP concentration, high fever, abdominal tenderness and foul smelling vaginal discharge) also showed no significant differences between the two races. In the current manuscript we distinguish unique pathways entailing maternal and fetal genetic variants associated with preterm birth.

**Table 1:** Differences between genotypic frequencies between African Americans and Caucasians

**Maternal DNA**	**Cases**	**Controls**
% Genotype frequency difference	68.5% (987/1440)	77.6% (1119/1442)
Fetal DNA		
% Genotype frequency difference	68.2% (984/1442)	80.2% (1156/1442)

Because the patterns of genetic association and therefore the detectable network patterns can be affected by differences in genotype and allele frequencies, it is important to assess the differences between our African-Americans and Caucasians prior to IPA analysis. After removing monomorphic markers and those with low genotyping efficiency from 1536 SNPs, 1442 remained for this analysis. Overall, the results indicate that the vast majority of SNPs assayed differed between African-Americans and Caucasians in genotype frequencies (~68–80%), and that controls appeared to be slightly more different than cases (Table 1). Of note, the vast majority of differences observed were highly significant. The proportion of comparisons with genotype frequency differences more significant than p = 0.001, range from 935 of 1442 SNPs in maternal controls to 668 of 1442 SNPs in fetal cases. These data demonstrate that the African-Americans and Caucasians were highly different in terms of the distribution of genetic variation at the candidate loci we studied. In addition, these proportions exceed the average across genome differences expected between Africans and Caucasians in the Hap Map data (average ~55% [data not shown]).

**Figure 1:**
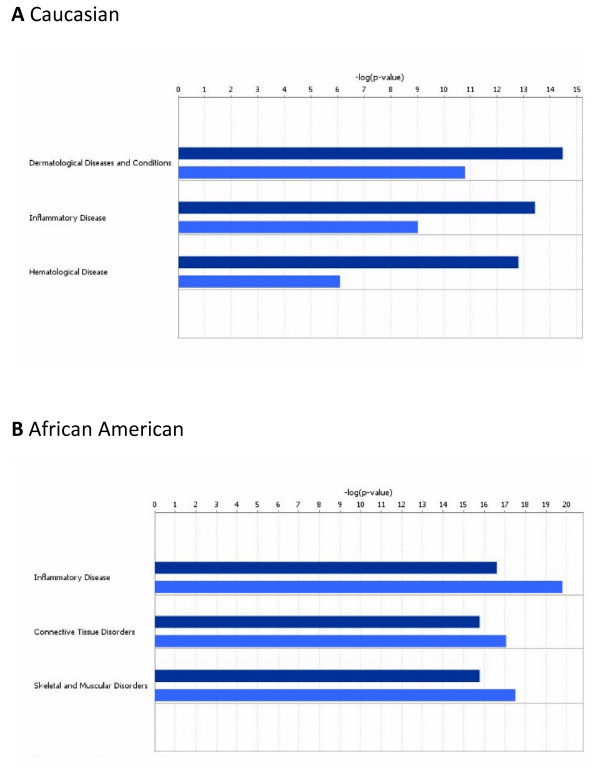
**Top disease and disorder functions determined by IPA to be overrepresented by focus genes**. Panel A, Caucasian mothers and their fetuses; panel B, African American mothers and their fetuses. Dark blue bars, maternal; light blue, fetal. The p-value for a given diseases and disorder annotation is calculated by the IPA software using Fishers Exact Test taking into account the number of focus genes that participate in that process in relation the total number of genes associated with that process in the IPA knowledgebase.

Based on the IPA curated disease ontology we determined which disease functions were most significantly associated with the aggregate of focus genes in each race. From all SNPs entered into the analysis, the IPA mapped 129 genes. Thus, disease function analysis defined a different set of genes for each group meeting the p ≤ 0.05 cutoff; specifically, 36 genes for Caucasian mothers, 39 genes for African American mothers, 35 genes for Caucasian fetuses, and 39 genes for African-American fetuses. These results indicate that the gene variants entered into our IPA analyses have been identified in the literature with a variety of diseases and disorders. Figure 1 shows the top three disease functions for each race as ranked by statistical significance that was calculated by the IPA functional analysis algorithm. Note that genes associated with inflammatory diseases are significant in both races, but that the p-values suggest greater significance in inflammation-associated genes in African-Americans than in Caucasians. Additionally, in the African Americans the fetal results are more significant than the maternal, but this is not the case in Caucasians. In general, the fetal results were more significant in African Americans than the maternal contribution for 13 higher order disease categories, but only 4 such categories in Caucasians.

Within the IPA disease ontology tree, the disease-related function contributing most to the significance was also of interest. For example, under hematological diseases, processes related to apoptosis and cell death were more significant in African-American mothers (p = 5.56E-12 to 8.11E-11) than in Caucasian mothers (1.07E-7 to 1.90 E-4). Conversely, hematological "disorders", thrombosis, and hemorrhage (also subsumed under hematological "diseases") were significant in Caucasian mothers (p-values 1.09E-6 to 1.73E-4) but did not reach significance in African American mothers.

**Figure 2:**
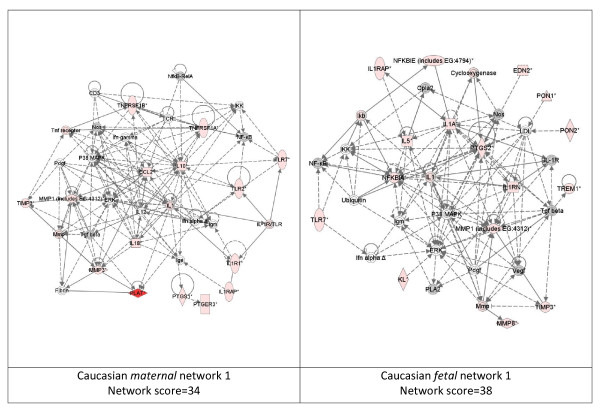
**Top ranking gene/protein networks determined by IPA analysis in Caucasian maternal (net work score 34) and fetal (net work score 38)**. Panel A, maternal networks; panel B, fetal networks. Solid lines show direct interaction (binding/physical contact); dashed line, indirect interaction supported by the literature but possibly involving one or more intermediate molecules that have not been investigated definitively. Molecular interactions involving only binding are connected with a solid line (no arrowhead) since directionality cannot be inferred. Focus genes: pink color, met criteria for case-control comparison for genotype at p ≤ 0.05; red, met criteria for case-control comparison for genotype at p ≤ 0.001; grey, indicating one or more SNP was analyzed in our data set but case-control comparison did not meet p ≤ 0.05; no color – additional interconnected genes generated algorithmically by IPA, i.e., proteins, or complexes, including new potential biomarkers. * indicates that there was more than one SNP probe for this gene tested and the most significant was placed into the analysis.

**Figure 3:**
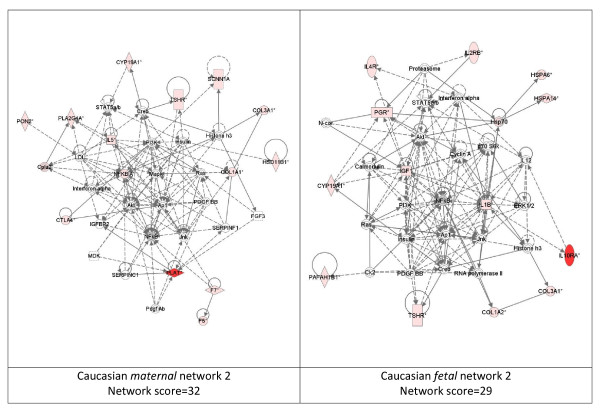
**Second ranking gene/protein networks determined by IPA analysis in Caucasian maternal (net work score 32) and fetal (net work score 29)**. Panel A, maternal networks; panel B, fetal networks. Solid lines show direct interaction (binding/physical contact); dashed line, indirect interaction supported by the literature but possibly involving one or more intermediate molecules that have not been investigated definitively. Molecular interactions involving only binding are connected with a solid line (no arrowhead) since directionality cannot be inferred. Focus genes: pink color, met criteria for case-control comparison for genotype at p ≤ 0.05; red, met criteria for case-control comparison for genotype at p ≤ 0.001; grey, indicating one or more SNP was analyzed in our data set but case-control comparison did not meet p ≤ 0.05; no color – additional interconnected genes generated algorithmically by IPA, i.e., proteins, or complexes, including new potential biomarkers. * indicates that there was more than one SNP probe for this gene tested and the most significant was placed into the analysis.

**Figure 4:**
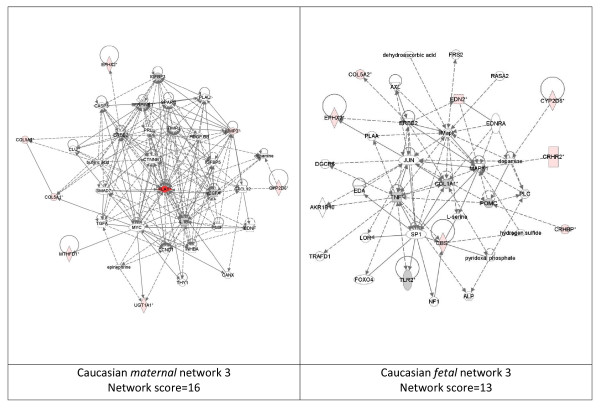
**Third ranking Gene/protein networks determined by IPA analysis in Caucasian maternal (net work score 16) and fetal (net work score 13)**. Panel A, maternal networks; panel B, fetal networks. Solid lines show direct interaction (binding/physical contact); dashed line, indirect interaction supported by the literature but possibly involving one or more intermediate molecules that have not been investigated definitively. Molecular interactions involving only binding are connected with a solid line (no arrowhead) since directionality cannot be inferred. Focus genes: pink color, met criteria for case-control comparison for genotype at p ≤ 0.05; red, met criteria for case-control comparison for genotype at p ≤ 0.001; grey, indicating one or more SNP was analyzed in our data set but case-control comparison did not meet p ≤ 0.05; no color – additional interconnected genes generated algorithmically by IPA, i.e., proteins, or complexes, including new potential biomarkers. * indicates that there was more than one SNP probe for this gene tested and the most significant was placed into the analysis.

**Figure 5:**
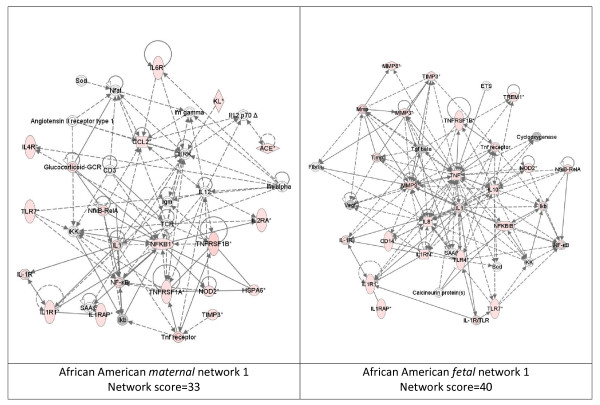
**Top ranking gene/protein networks determined by IPA analysis in African Americans maternal (net work score 33) and fetal (net work score 40)**. Panel A, maternal networks; panel B, fetal networks. Solid lines show direct interaction (binding/physical contact); dashed line, indirect interaction supported by the literature but possibly involving one or more intermediate molecules that have not been investigated definitively. Molecular interactions involving only binding are connected with a solid line (no arrowhead) since directionality cannot be inferred. Focus genes: pink color, met criteria for case-control comparison for genotype at p ≤ 0.05; red, met criteria for case-control comparison for genotype at p ≤ 0.001; grey, indicating one or more SNP was analyzed in our data set but case-control comparison did not meet p ≤ 0.05; no color – additional interconnected genes generated algorithmically by IPA, i.e., proteins, or complexes, including new potential biomarkers. * indicates that there was more than one SNP probe for this gene tested and the most significant was placed into the analysis.

**Figure 6:**
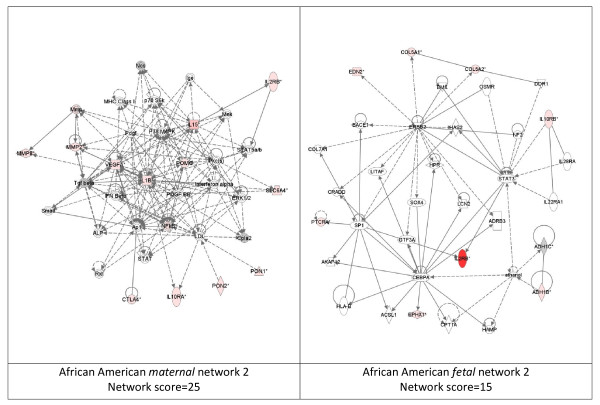
**Second ranking gene/protein networks determined by IPA analysis in African Americans maternal (net work score 25) and fetal (net work score 15)**. Panel A, maternal networks; panel B, fetal networks. Solid lines show direct interaction (binding/physical contact); dashed line, indirect interaction supported by the literature but possibly involving one or more intermediate molecules that have not been investigated definitively. Molecular interactions involving only binding are connected with a solid line (no arrowhead) since directionality cannot be inferred. Focus genes: pink color, met criteria for case-control comparison for genotype at p ≤ 0.05; red, met criteria for case-control comparison for genotype at p ≤ 0.001; grey, indicating one or more SNP was analyzed in our data set but case-control comparison did not meet p ≤ 0.05; no color – additional interconnected genes generated algorithmically by IPA, i.e., proteins, or complexes, including new potential biomarkers. * indicates that there was more than one SNP probe for this gene tested and the most significant was placed into the analysis.

**Figure 7:**
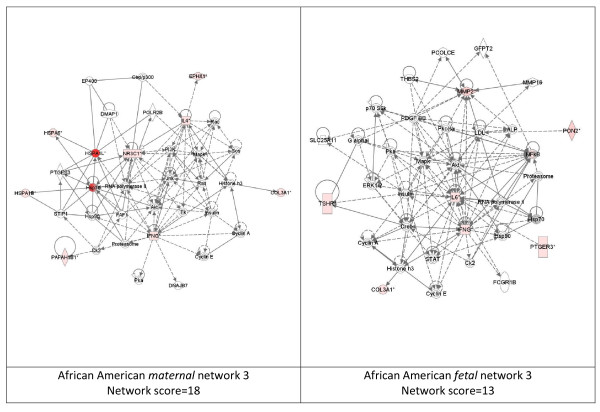
**Third ranking Gene/protein networks determined by IPA analysis in Caucasian maternal (net work score 18) and fetal (net work score 13)**. Panel A, maternal networks; panel B, fetal networks. Solid lines show direct interaction (binding/physical contact); dashed line, indirect interaction supported by the literature but possibly involving one or more intermediate molecules that have not been investigated definitively. Molecular interactions involving only binding are connected with a solid line (no arrowhead) since directionality cannot be inferred. Focus genes: pink color, met criteria for case-control comparison for genotype at p ≤ 0.05; red, met criteria for case-control comparison for genotype at p ≤ 0.001; grey, indicating one or more SNP was analyzed in our data set but case-control comparison did not meet p ≤ 0.05; no color – additional interconnected genes generated algorithmically by IPA, i.e., proteins, or complexes, including new potential biomarkers. * indicates that there was more than one SNP probe for this gene tested and the most significant was placed into the analysis.

**Figure 8:**
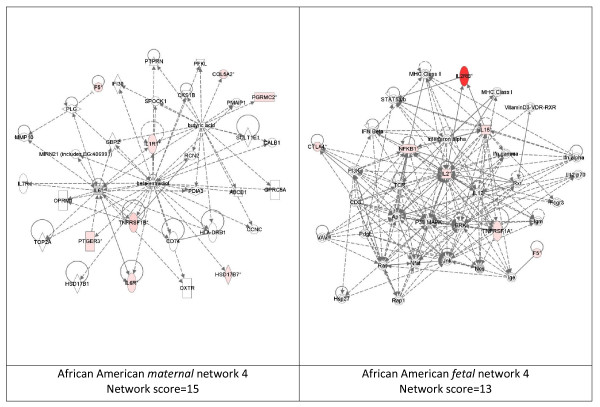
**Fourth ranking Gene/protein networks determined by IPA analysis in Caucasian maternal (net work score 15) and fetal (net work score 13)**. Panel A, maternal networks; panel B, fetal networks. Solid lines show direct interaction (binding/physical contact); dashed line, indirect interaction supported by the literature but possibly involving one or more intermediate molecules that have not been investigated definitively. Molecular interactions involving only binding are connected with a solid line (no arrowhead) since directionality cannot be inferred. Focus genes: pink color, met criteria for case-control comparison for genotype at p ≤ 0.05; red, met criteria for case-control comparison for genotype at p ≤ 0.001; grey, indicating one or more SNP was analyzed in our data set but case-control comparison did not meet p ≤ 0.05; no color – additional interconnected genes generated algorithmically by IPA, i.e., proteins, or complexes, including new potential biomarkers. * indicates that there was more than one SNP probe for this gene tested and the most significant was placed into the analysis.

**Figure 9:**
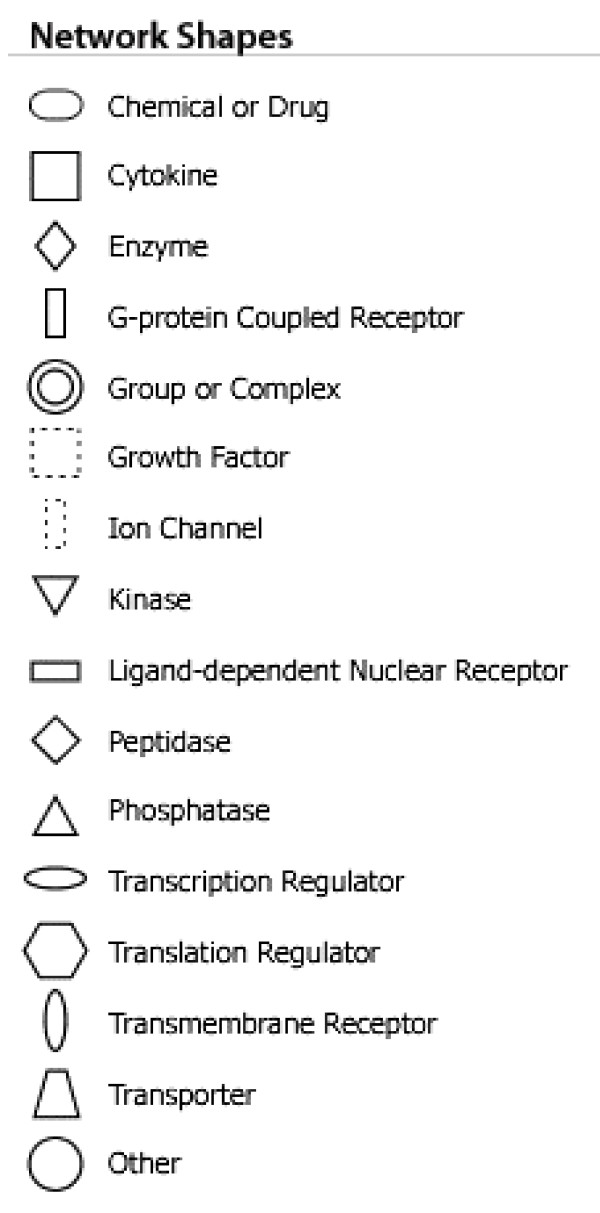
**Legends used in network diagrams (figures 2–8)**.

While these analyses revealed disease functions assigned to our input genes, we were also interested in the functional biological networks employing these genes and proteins. (Figures 2, 3, 4, 5, 6, 7, 8, 9) Using the IPA network algorithm, we constructed a series of connectivity maps derived from millions of molecular interactions and regulatory processes housed in the IPA knowledgebase (Figures 2 through 8). These networks were generated based on their composite score, which represents the negative log of the p-value for the likelihood these molecules would be found together by chance. Accordingly, a higher score indicates greater statistical significance that molecules depicted in the network are interconnected. Each network is a graphical representation of the molecular relationships between genes/gene products. Genes or gene products are represented as nodes, and the biological relationship between two nodes is represented as an edge (line). All edges are supported by at least 1 reference from the literature, from a textbook, or from canonical information stored in the Ingenuity Pathways Knowledge Base. For Caucasian mothers, IPA generated three highly significant networks (scores 16 to 34), and Caucasian fetuses had a different set of three networks (scores 13 to 38). For African-American mothers there were four highly significant networks (scores 15 to 33), and African-American fetuses had another four networks (scores 13 to 40). Note each network includes a number of "partner" molecules that were assigned to the network by the IPA algorithm, but which were not among the input genes.

## Discussion

Biological pathways leading to preterm birth are intertwined and redundant, and can involve inflammation, collagen remodeling, decidual vascular insufficiency, and myometrial contraction. Thus, parturition represents a tuned orchestration of regulatory loops. Therefore, in this study we examined the hypothesis that genetic variations in the molecules involved in these regulatory networks are important determinants of preterm birth. Our finding that preterm birth susceptibility genes mapped to multiple physiological functions including inflammatory diseases, dermatologic and connective tissue disorders (i.e. collagen metabolism disorders), hematological disease (such as decidual hemorrhage), and skeletal and muscular disorders (i.e. involving inflammation, collagen, and muscle function) supports the role of multiple physiological processes in preterm birth (Table 2, 3, 4). Importantly, the relative relationships/roles of these processes differed by race. Specifically, different mechanistic pathways appeared to be more common in the pathophysiology of preterm birth in African Americans and Caucasians, as suggested by the differences in the IPA scores for disease functions between the two races (Table 2, 3). Among African-Americans the prominent disease functions detected involved inflammation, connective tissue disorders and skeletal and muscle disorders, whereas in Caucasians dermatological diseases and conditions (involving collagen metabolism), inflammatory diseases, and hematological diseases were most prominent. Tables 2,3 and 4 specifies the genes associated with the top-ranked disease and disorder functions in our IPA analysis, and provides the context of these results with respect to preterm birth. Further, while there was evidence for maternal and fetal genetic contribution to preterm birth risk in both races, the relative roles of maternal and fetal variants to the disease functions was different in the two races. In African-Americans the fetal genetic variants were more prevalent, but in Caucasians the maternal disease functions showed more significance. We conclude that based on specific risk exposure (initiators of preterm labor process) and an individual's own genetic constitution the underlying cellular and molecular pathways that lead to preterm birth may be more or less commonly depending on race.

**Table 2:** Disease functions as detected by IPA based on significant genetic variations in maternal and fetal candidate genes in Caucasians and its interpretation in spontaneous preterm birth.

**Disease functions as detected by IPA**	**Genes involved in determining the disease functions**		**Potential pathophysiological role in preterm birth**
	**Mother**	**Fetus**	
**Dermatologic disease and conditions**	COL1A1, COL3A1, COL5A1, COL5A2, CTLA4, CYP19A1, IL5, IL10, IL18, MMP3, MMP1, NFKBIA, PLAT, PTGS1, TLR2, TLR7, TNFR1, TNFR2,	COL1A2, COL3A1, COL5A2, CYP19A1, IGF1, IL5, IL1A, IL1B, IL1RN, IL4R, MMP, NFKBIA, PGR, PTGS2, TLR7	Because dermatological disorders typically involve combined collagen remodeling aided by inflammation and, this function appears to reflect premature cervical ripening and membrane weakening related to preterm birth. Collagenolysis may involve an underlying inflammatory process as suggested by cytokines, cytokine receptors, cytokine signaling pathway genes that is likely a secondary event. Etiology → Collagenolysis → inflammation → Premature cervical ripening and membrane weakening → Preterm birth.
**Inflammatory disease**	CCL2, CTLA4, CYP19A1, F7, IL5, IL10, IL18, IL1R1, MMP3, MMP1, NFKBIA, PLA2G4A, PLAT, PTGER3, PTGS1, TIMP3, TLR2, TNFR1, TNFR2	CRHR2, CYP19A1, IGF1, IL5, IL10RA, IL1A, IL1B, IL1RN, IL2RB, IL4R, KL, MMP8, MMP1, NFKBIA, PGR, PTGS2, TIMP3, TREM1	Underlying inflammatory conditions associated with preterm birth (see above)
**Hematological disease**	CCL2, CTLA4, CYP19A1, F5, F7, HSD11B1, IL5, IL10, IL18, IL1RAP, NFKBIA, PLA2G4A, PLAT, PTGS1, TLR2, TNFR1, TNFR2	CBS, IGF1, IL5, IL1A, IL1B, IL1RAP, IL1RN, IL2RB, IL4R, MMP8, NFKBIA, NFKBIE, PGR, PTGS2	Involvement of genes in the hematological pathway indicates decidual hemorrhage and associated problems associated with Caucasian preterm birth.

**Table 3:** Disease functions as detected by IPA based on significant genetic variations in maternal and fetal candidate genes in African Americans and its interpretation in spontaneous preterm birth.

**Disease functions as detected by IPA**	**Genes involved in determining the disease functions**		**Potential pathophysiological role in preterm birth**
	**Mother**	**Fetus**	
**Inflammatory disease**	ACE, CCL2, CTLA4, IFNG, IL4, IL15, IL10RA, IL1B, IL1R1, IL2RA, IL2RB, IL4R, IL6R, KL, MMP2, MMP8, NFKB1, NOD2, NR3C1, POMC, PTGER3, SLC6A4, TIMP3, TNFR1, TNFR2, VEGFA	CD14, CTLA4, IFNG, IL2, IL6, IL8, IL10, IL15, IL10RB, IL1R1, IL1RN, IL2RB, MMP2, MMP3, MMP8, MMP9, NFKB1, NFKBIB, NOD2, PTGER3, TIMP3, TLR4, TNF, TNFR1, TNFR2, TREM1	Overwhelming inflammation appears to be the primary effecter of preterm birth in African Americans regardless of etiology. Fetal response is dominant (see Figure 2) along with complementary maternal contributions. This combined inflammatory process that can activate multitudes of other pathways seem primary disease function resulting in preterm birth Etiology → Inflammation → Activation of multitudes of uterotonic events → Preterm birth
**Connective tissue disease**	CCL2, IFNG, IL4, IL15, IL10RA, IL1B, IL1R1, IL2RB, IL4R, IL6R, MMP2, NFKB1, NR3C1, POMC, SLC6A4, TIMP3, TNFR1, NFR2,	IFNG, IL6, IL8, IL10, IL15, IL1R1, IL1RN, IL2RB, MMP2, MMP3, MMP9, NFKB1, TIMP3, TLR4, TNF, TNFR1, TNFR2	This function also involves immune and inflammatory response genes. Matrix metalloproteinases can function in inflammation as well as collagen turnover function; e.g. involved in cervical ripening and membrane matrix degradation.
**Skeletal and muscular disorder**	CCL2, CTLA4, IFNG, IL4, IL15, IL10RA, IL1B, IL1R1, IL2RB, IL4R, IL6R, KL, MMP2, NFKB1, NOD2, NR3C1, POMC, SLC6A4, TNFR1, TNFR2, VEGFA	CD14, CTLA4, IFNG, IL2, IL6, IL8, IL10, IL15, IL1R1, IL1RN, IL2RB, MMP2, MMP3, MMP9, NFKB1, NFKBIB, NOD2, TLR4, TNF, TNFR1, TNFR2, TSHR	Most of these genes function in inflammatory regulation are related to rheumatic disorders, including matrix metalloproteinases that are also involved in cervical ripening and membrane matrix degradation.

**Table 4:** Gene symbols and their names from Table 2 and 3

**Gene symbol**	**Name**
CBS	Cystathionine-beta-synthase
CCL2	Chemokine (C-C motif) ligand 2
CD14	Cluster of differentiation 14
COL1A1	Collagen, type I, alpha 1
COL3A1	Collagen, type III, alpha 1
COL5A1	Collagen, type v, alpha 1
COL5A2	Collagen, type v, alpha 2
CRHR2	Corticotropin releasing hormone receptor 2
CTLA4	Cytotoxic T-Lymphocyte Antigen 4
CYP19A1	Cytochrome P450, family 19, subfamily A, polypeptide 1
F5	FA Factor 5
F7	FA Factor 7
IFNG	Interferon gamma
IGF1	Insulin growth factor 1
IL10	Interleukin-10
IL10RA	IL-10 receptor antagonist
IL15	Interleukin 15
IL18	Interleukin-18
IL1A	Interleukin-1 α
IL1B	Interleukin-1 β
IL1R1	IL-1 receptor 1
IL1RAP	IL-1 receptor accessory protein
IL1RN	IL-1 receptor antagonist
IL2RB	IL-2 receptor β
IL4R	Interleukin-4 receptor
IL5	Interleukin-5
IL-6	Interleukin 6
KL	KLOTHO type-I membrane protein
MMP1	Matrix metalloproteinase 1/collagensase 1
MMP2	Matrix metalloproteinase 2/Glatinase A
MMP3	Matrix metalloproteinase 3, stromelysin 1
MMP8	Matrix metalloproteinase 8, neutrophil collagenase
MMP9	matrix metalloproteinase 9/Gelatinase B
NFKBIA	nuclear factor of kappa light polypeptide gene enhancer in B-cells inhibitor, alpha
NFKBIB	Nuclear factor of kappa light polypeptide gene enhancer in B-cells inhibitor, beta,
NFKBIE	Nuclear factor of kappa light polypeptide gene enhancer in B-cells inhibitor, epsilon
NOD2	Nucleotide-binding oligomerization domain containing 2
NR3C1	Nuclear receptor subfamily 3
PGR	Progesterone receptor
PLA2G4A	Phospholipase A2, group IVA
PLAT	Tissue plasminogen activator
POMC	Proopiomelanocortin
PTGER3	Prostaglandin E receptor 3
PTGS1	Prostaglandin-endoperoxide synthase 1
PTGS2	Prostaglandin-endoperoxide synthase 2
SLC6A4	Sodium-dependent serotonin transporter
TIMP3	Tissue inhibitor of matrix metalloproteinase 3
TLR2	Toll like receptor 2
TLR4	Toll like receptor 4
TLR7	Toll like receptor 7
TNF	Tumor necrosis factor
TNFR1	Tumor necrosis factor receptor 1
TNFR2	Tumor necrosis factor receptor 2
TREM1	Triggering receptors on myeloid cell -1
TSHR	Thyroid stimulating hormone receptor
VEGFA	Vascular endothelial growth factor A

In this study molecular groupings generated by IPA suggest a new way of examining SNP data to predict events involved in preterm birth. The networks that were found to be most significant (statistically) by IPA can be viewed as modules of interacting molecules that act in key physiological compartments involved in the pathophysiology of preterm birth. For each network, the IPA analysis generated new potential interacting biomarkers (partner molecules) that are highly interconnected with the variant genes we tested and provide a set of new testable hypotheses regarding predisposition to preterm birth.

Based on candidate gene studies, our group has already reported clear disparity in genetic associations with preterm birth in both maternal and fetal genome between African-Americans and Caucasians [10,13,14]. However, such studies provide little information on biological relevance other than the most simplistic relationships between single genes and PTB. The present study differs in that it provides a large set of potential risk factors that can ultimately predict risk based on gene-gene and gene-environment interactions based on documented biological pathways. The IPA approach provides more insight into potential functional roles of genes and more testable and biologically plausible hypotheses. Therefore, Based we can now argue with increased confidence that genetic variants linked with preterm birth participate in discrete pathways that define specific disease functions in each race.

This systems biology approach enables the identification of new pathways and genes. Understanding these disease functions and molecular networking can help categorize women into specific risk groups for targeted intervention. This approach introduces few assumptions concerning the types of pathophysiological processes that might be discerned. Accordingly, some "disease functions" identified by IPA as being associated with preterm birth may not have an obvious connection with parturition or preterm birth. However, the strength of Ingenuity is its broad and unbiased coverage of peer reviewed literature in cell biology, organ physiology, and pathology. Thus, the literature in diverse fields may be used to reveal clues to preterm birth, and the disease functions, it can provide substantial insight as long as caution is used in interpreting the disease functions output from IPA. We also emphasize that the primary data on which these analyses are based is small and will also require follow-up studies.

As with all studies connecting genetics, race, and PTB, our findings could be misused as an excuse for failing to rectify inequalities in social conditions and healthcare delivery that have well established links with PTB. However, genes act within environmental context, and we do not suggest race can be used as a proxy for genetic markers. For example, genes controlling inflammatory pathways are related to susceptibility to bacterial vaginosis, autoimmune disorders [7], and depression [28]. If our data indicates that some mothers have vulnerabilities in these pathways, this implies a need for greater emphasis on preventive measures to improve the socio-environmental conditions that bring about these conditions.

In conclusion, we performed a network analysis to identify molecules and risk pathways that were not explicitly tested in our original genetic studies. These analyses produced statistical and visual evidence of racial disparity in key networks associated with other disease phenotypes that can provide important insights into the pathophysiology of preterm birth.

## Competing interests

The authors declare that they have no competing interests.

## Authors' contributions

RM Conceptualized using bioinformatics tools to the genetic association data set, analysis and interpretation of analysis, drafted the manuscript. BP performed the ingenuity based data analysis and assisted manuscript preparation. DV performed genetic data analysis. SMW assisted and advised on genetic data analysis and assisted in manuscript preparation. MM assisted in concept development and manuscript preparation. SJF assisted in recruitment of subjects, provided clinical impact of data, assisted manuscript preparation. PT assisted in bringing the team to perform this study, analysis and assisted in manuscript preparation.
